# Possible role of *Porphyromonas gingivalis* in orodigestive cancers

**DOI:** 10.1080/20002297.2018.1563410

**Published:** 2019-01-09

**Authors:** Ingar Olsen, Özlem Yilmaz

**Affiliations:** a Department of Oral Biology, Faculty of Dentistry, University of Oslo, Oslo, Norway; b Department of Oral Health Sciences, Medical University of South Carolina, Charleston, SC, USA

**Keywords:** *Porphyromonas gingivalis*, orodigestive cancers, oral cavity, esophagus, colon, pancreas, precancerous lesions, experimental models, direct relationship

## Abstract

There is increasing evidence for an association between periodontitis/tooth loss and oral, gastrointestinal, and pancreatic cancers. Periodontal disease, which is characterized by chronic inflammation and microbial dysbiosis, is a significant risk factor for orodigestive carcinogenesis. *Porphyromonas gingivalis* is proposed as a keystone pathogen in chronic periodontitis causing both dysbiosis and discordant immune response. The present review focuses on the growing recognition of a relationship between *P. gingivalis* and orodigestive cancers. *Porphyromonas gingivalis* has been recovered in abundance from oral squamous cell carcinoma (OSCC). Recently established tumorigenesis models have indicated a direct relationship between *P. gingivalis* and carcinogenesis. The bacterium upregulates specific receptors on OSCC cells and keratinocytes, induces epithelial-to-mesenchymal (EMT) transition of normal oral epithelial cells and activates metalloproteinase-9 and interleukin-8 in cultures of the carcinoma cells. In addition, *P. gingivalis* accelerates cell cycling and suppresses apoptosis in cultures of primary oral epithelial cells. In oral cancer cells, the cell cycle is arrested and there is no effect on apoptosis, but macro autophagy is increased. *Porphyromonas gingivalis* promotes distant metastasis and chemoresistance to anti-cancer agents and accelerates proliferation of oral tumor cells by affecting gene expression of defensins, by peptidyl-arginine deiminase and noncanonical activation of β-catenin. The pathogen also converts ethanol to the carcinogenic intermediate acetaldehyde. In addition, *P. gingivalis* can be implicated in precancerous gastric and colon lesions, esophageal squamous cell carcinoma, head and neck (larynx, throat, lip, mouth and salivary glands) carcinoma, and pancreatic cancer. The fact that distant organs can be involved clearly emphasizes that *P. gingivalis* has systemic tumorigenic effects in addition to the local effects in its native territory, the oral cavity. Although coinfection with other bacteria, viruses, and fungi occurs in periodontitis, *P. gingivalis* relates to cancer even in absence of periodontitis. Thus, there may be a direct relationship between *P. gingivalis* and orodigestive cancers.

## Introduction

Over the years, a number of bacteria have been associated with periodontitis [1]. Among them, *Porphyromonas gingivalis* is regarded as a keystone pathogen in adult periodontitis [2–9]. This bacterium has also been associated with a number of extraoral infection-related diseases, for example, cardiovascular diseases, diabetes, preterm birth, pulmonary disease, and rheumatoid arthritis [10,11]. In *P. gingivalis*, the most important virulence factors are lipopolysaccharide (LPS), fimbriae, gingipains and outer membrane vesicles. Major pathogenic mechanisms include ability to create a dysbiotic microbiota and a dysregulated immune defense [12–18]. It is also noteworthy that *P. gingivalis* can invade oral epithelial and endothelial cells [19–21] and induce potent production of pro-inflammatory cytokines [22].

**Figure 1.**
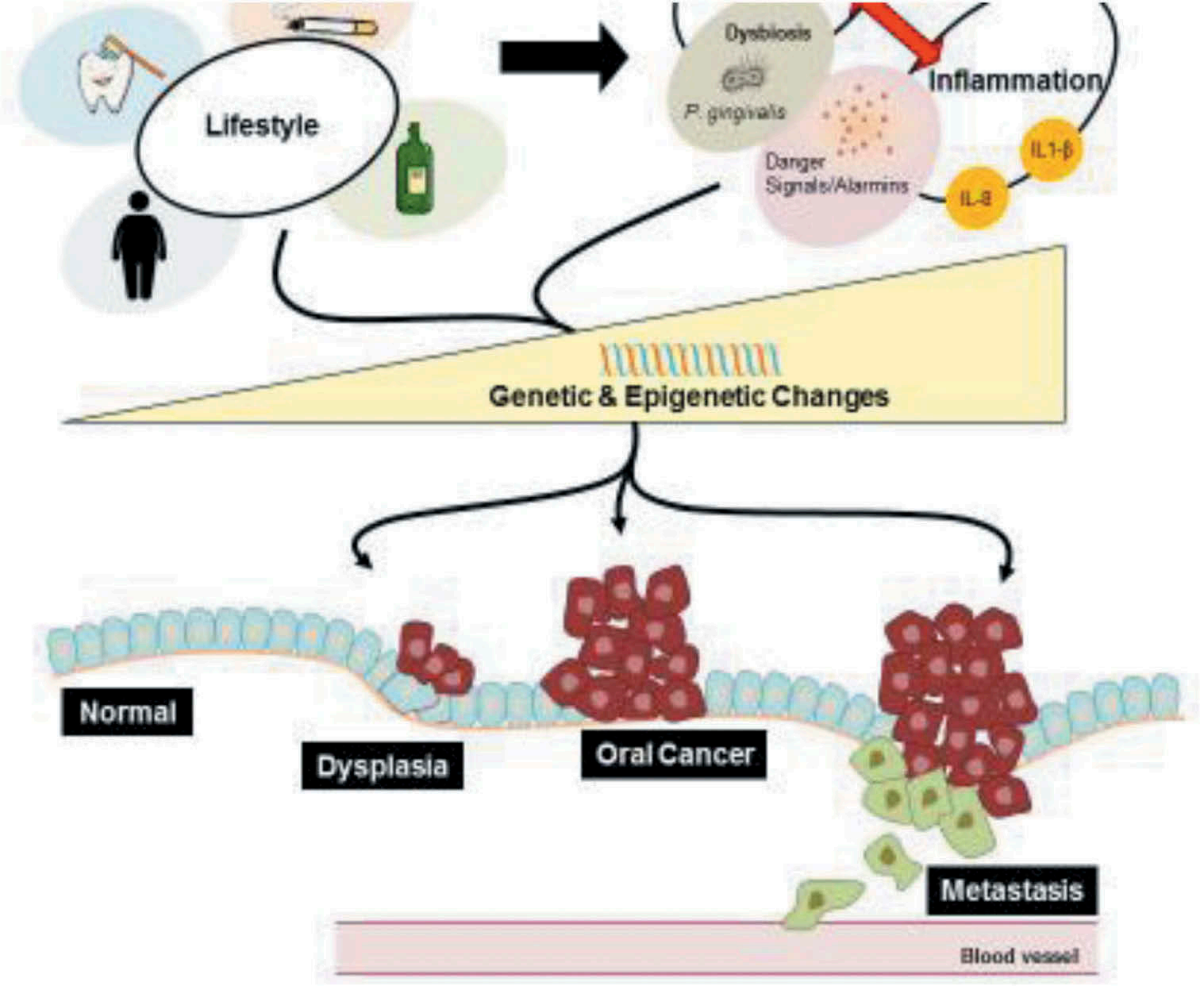
Factors contributing to oral cancer. Unhealthy lifestyle choices such as smoking, consumption of alcohol, obesity, and poor oral hygiene increase the incidence of periodontal disease and inflammation in the oral mucosa. *Porphyromonas gingivalis*, as a major contributor in the etiology of periodontal disease, is a keystone pathogen, which facilitates dysbiosis, and can also invade epithelial cells and modify the cellular environment intra-and extra-cellularly. Additionally, the increase of danger signals, such as ATP and other alarmins, and release of pro-inflammatory cytokines and chemokines create a chronic inflammatory state in the oral cavity associated with periodontal disease. Microbial factors, chronic inflammation associated with periodontal disease, and general poor lifestyle choices induce genetic and epigenetic changes in the cell, which may promote orodigestive cancer development and progression.

**Figure 2.**
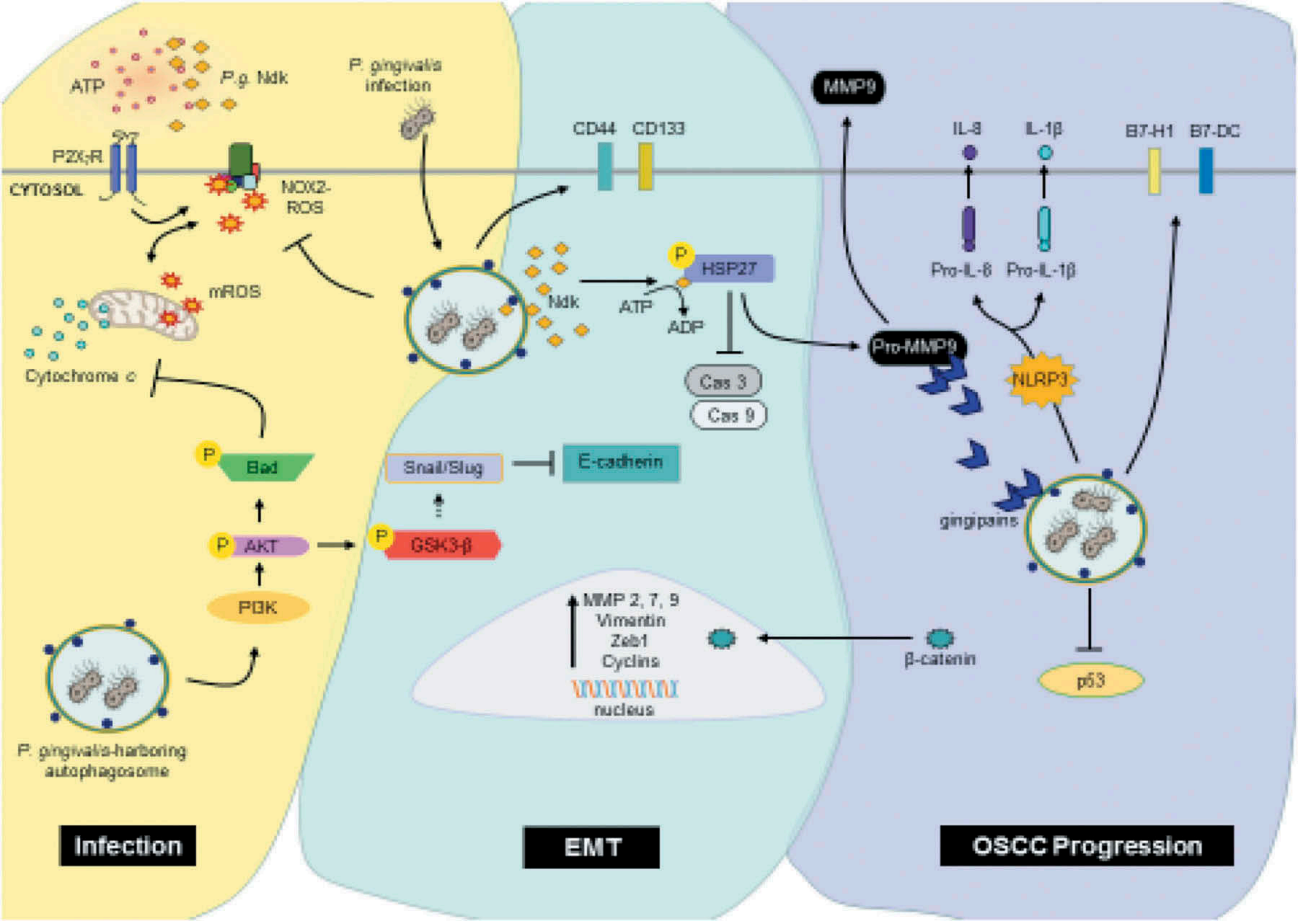
*Porphyromonas gingivalis* promoting the development and progression of OSCC. *Infection*: Intracellular *Porphyromonas gingivalis* infection promotes survival and proliferation of the epithelial cell by increasing PI3K/Akt signaling shortly after infection, resulting in the inhibition of intrinsic apoptosis. Additionally, through secretion of its effector protein, nucleoside diphosphate kinase (NDK), *P. gingivalis* blocks extracellular ATP/P2X7 danger signaling, protecting itself and the host epithelial cell from damaging mitochondrial and NOX2 generated ROS. *EMT*: The epithelial-mesenchymal transition (EMT) is promoted through the inactivation of GSK3-β, which facilitates a switch from E-cadherin to Vimentin via increased expression and availability of Snail, Slug and β-catenin transcription factors. β-catenin also upregulates cyclins, ZEB1, and MMPs, resulting in increased epithelial cell proliferation and migration. *Porphyromonas gingivalis* continues to promote EMT through direct phosphorylation of HSP27 via its effector protein NDK, leading to increased levels of pro-MMP9. Furthermore, *P. gingivalis* increases the expression of cancer stem cell markers CD44 and CD133. *OSCC Progression: P. gingivalis* further maintains a pro-survival and proliferative phenotype in cancer cells by blocking p53. An invasive phenotype is promoted through gingipains – key virulence factors of *P. gingivalis* – which bind and process pro-MMP9 to MMP9. Moreover, *P. gingivalis* modulates the immune environment through cytokine and chemokine secretion and the increased expression of B7-H1 and B7-DC receptors which cause T-cell anergy and apoptosis of activated T cells.

Increasing evidence implicates *P. gingivalis* in the etiology of oral, gastrointestinal, and pancreatic cancers [23]. Interestingly, Ahn et al. [24] found that orodigestive cancer mortality is related to periodontitis and to serum *P. gingivalis* IgG, independent of periodontal disease. This indicates that *P. gingivalis* can have an important role in the development of orodigestive carcinogenesis irrespective of periodontitis. A review of the emerging role of bacteria in oral carcinogenesis was recently published by Perera et al. [25]. The present review aims to systematically broaden our most recent understanding of the relationship between *P. gingivalis* and orodigestive cancers (Figures 1 and 2).

## Oral cancer

Squamous cell carcinomas (SCCs) constitute more than 90% of oral cancers, listing among the top 10 most common types of cancer worldwide [26–29]. An estimated rate of 350,000–400,000 new cases worldwide are diagnosed each year [29].

### Relationship between periodontal disease/tooth loss and orodigestive cancer

Several epidemiological and clinical studies have found a positive relationship between periodontal disease or tooth loss and the progression of cancers such as oral cancer, gastric cancer, pancreatic cancer, and even gastric precancerous lesions [29–39]. In a study where meta-analysis was applied, patients with periodontitis had a 2.66-fold higher risk for developing oral cancer, and periodontitis was an independent risk indicator [40].

### Abundance of Porphyromonas gingivalis in gingival squamous cell carcinoma

*Porphyromonas gingivalis* occurred in significantly higher levels in sampled gingival SCCs than in normal gingival tissue (*p* < 0.05) [41]. It was also abundant in paraffin-embedded samples taken from cancerous tissue, indicating a potential relationship with gingival squamous cells carcinoma. This was in contrast to *Streptococcus gordonii*, which is a non-invasive oral bacterium [41]. No information on comorbidity factors was given and it was not clear whether host cell invasion of *P. gingivalis* was before or after cancerogenic transformation of cells.

### Porphyromonas gingivalis affects carcinogenesis in animal models

In a newly established murine model for periodontitis-associated oral tumorigenesis, it was demonstrated that chronic infection induced by *P. gingivalis* and *Fusobacterium nucleatum*, together with administration of the oral carcinogen 4-nitroquinoline-1-oxide (4NQO), promoted transformation of oral squamous carcinogenic cells and increased signaling along the interleukin-6 (IL-6)-signal transducer and activator of transcription 3 (STAT3) axis [32]. This in turn induced effectors driving growth and invasiveness of cells from OSCC. The findings agreed with other reports showing that IL-6 signaling and STAT3 activity are pro-tumorigenic [42,43]. The production of IL-6 in epithelial cells was increased by *P. gingivalis* activating the Janus kinase 2 (JAK2) and Glycogen synthase kinase 3 beta (GSK3β) pathways [42,44]. Results by Binder Gallimidi et al. [32] indicated that *P. gingivalis* and *F. nucleatum* stimulate tumorigenesis by direct interaction with oral epithelial cells through activation of Toll-like-receptors (TLRs) causing IL-6 production from the epithelial cells. In addition, oral pathogens stimulated proliferation of OSCC and expression of key molecules in tumorigenesis such as cyclin D1, Matrix Metalloproteinase-9 (MMP-9) and heparanase [32].

Geng et al. [45] established another *in vitro* model exposing human immortalized oral epithelial cells to *P. gingivalis* at a low multiplicity of infection for 5–23 weeks. Persistent exposure to *P. gingivalis* induced cell morphological changes, increased the proliferation ability with a higher S phase fraction in the cell cycle, and promoted cell migration and invasion. It was suggested that tumor-related genes such as *NNMT*, *FLI1*, *GAS6*, *IncRNA CCAT1*, *PDCD1LG2*, and *CD274* are key regulators in tumor-like transformation under long-time exposure to *P. gingivalis*. Thus, chronic infection with *P. gingivalis* may be regarded as a potential risk factor for oral cancer.

### Porphyromonas gingivalis upregulates receptors on squamous carcinoma cells and gingival keratinocytes

Expression of B7 homolog 1 (B7-H1) receptors occurs in most human cancers causing anergy and apoptosis of activated T cells. The B7-H1 receptors are upregulated on cancer/transformed cells, and the B7-H1 receptors then interfere with PD1 receptors on tumor-infiltrating lymphocytes (TILS), blocking the PD1 receptors’ cytotoxic activity against the cancerous epithelial cells. This may enable tumor cells to overcome the host response. After infection with two separate *P. gingivalis* strains (W83 and ATCC 33277^T^), both B7-H1 and B7-DC receptors were upregulated on oral squamous carcinoma cells, SCC-25, and BHY, and on human primary gingival epithelial cells [46]. After staining with specific antibodies against human B7-H1 and B7-DC, mean fluorescence intensity increased significantly from 4.5 to 9.9 for B7-H1 and from 6.9 to 15.0 for B7-DC in SCC-25 cells. Similar effects were seen in human primary gingival epithelial cells. In comparison, the commensal bacterium *S*. *salivarius* K12 did not cause upregulation. However, the implementation of *S. salivarius* K12, a nonpathogenic oral commensal probiotic bacterium, as a sole control microorganism rather stands incomplete. Additional inclusion of pathogenic and/or opportunistic oral microorganisms that are also Gram-negative is warranted to challenge and substantiate the physiological importance and specificity of these findings.

In culture experiments, the membrane fraction of *P. gingivalis* caused upregulation of the immune-regulatory receptor PD-L1 (B7-H1) in oral squamous carcinoma cells and gingival epithelial cells [47]. The total membrane fraction produced the highest upregulation in B7-H1, followed by the outer and inner membrane. It also seems that B7-H1 expression may contribute to evasion of the immune response, which could lead to chronicity of *P. gingivalis* infection.

### Porphyromonas gingivalis induces epithelial-to-mesenchymal transition of human primary oral epithelial cells and oral squamous cell carcinoma (OSCC) cells

In order to mimic chronic irritation by *P. gingivalis* in the mouth, OSCC cells were exposed to *P. gingivalis* twice a week for 5 weeks [48]. The exposure resulted in elongated morphology of host cancer cells with decreased expression of specific epithelial markers, indicating EMT transition. In addition, migratory and invasive properties of the OSCC were revealed. There was also an increased expression of CD44 and CD133, which are cancer stem cell markers.

An important regulator of EMT, phospho-GSK3ß, was found to increase significantly (*p* < 0.01) in human primary epithelial cells during the course of *P. gingivalis* infection [49]. Simultaneously, EMT-associated transcription factors such as Slug, Snail, and ZEB1 (Zinc finger E-box-binding homeobox 1) increased significantly (*p* < 0.01) in protein and mRNA expression. The protein expression mesenchymal intermediate filament, vimentin, was significantly enhanced over the 120-h period of infection with *P. gingivalis*. When the adhesion molecule E-cadherin was analyzed, a significant decrease (*p* < 0.05) in expression and loss of membrane localization together with ß-catenin were seen in the oral epithelial cells. In contrast, MMP-2, −7 and −9 were markedly increased during long-term infection. *Porphyromonas gingivalis* infection also promoted migration of *P. gingivalis*-infected cells. In contrast, there was only a slight increase following coinfection with *F. nucleatum* [49]. Accordingly, human primary oral epithelial cells acquired initial molecular and cellular changes consistent with EMT induction during long-term infection with *P. gingivalis* [49]. While the sustained levels of *P. gingivalis* survival in the primary epithelial cells strongly correlated with the aforementioned phenotypic changes, it would be interesting to see whether bacterial secreted effectors such as nucleoside-diphosphate-kinase (NDK) or structural virulence factors such as fimbriae and LPS could contribute to EMT independently [50]. This is especially interesting because these properties have been shown to contribute to bacterial immune evasion as well as bacterial persistence in epithelial cells [23,51,52].

Another study, however, suggested that EMT was induced in OSCC cells following stimulation with both *P. gingivalis* and *F. nucleatum* [53]. There was a significant upregulation after 1, 5 and 8 days in the transcription of mesenchymal markers of EMT and a downregulation of epithelial markers compared to unstimulated controls. All cytokines examined such as Transforming growth factor beta 1 (TGF-ß1) and tumor necrosis factor alpha (TNF-α) were significantly increased by the periodontal pathogens. The cytokines were suggested to be involved in EMT-induction and the transcription factor Zinc finger protein SNAI1 (Snail) activation [53]. The downregulation of epithelial markers caused a significant decrease in impedance resistance of cell monolayers towards passage of electrical current in cultured epithelial cell layers. Accordingly, both these periodontal pathogens caused changes in OSCC at molecular, structural, and behavioral levels in conformation with EMT.

Sztukowska et al. [54] found that infection of immortalized gingival epithelial cells (TIGK cells) with *P. gingivalis* induced expression and nuclear localization of the ZEB1 transcription factor that controls EMT. *Porphyromonas gingivalis* strains that lacked the FimA fimbrial protein had reduced ability to induce ZEB1 expression. Increased expression of ZEB1 was also caused by *P. gingivalis* in a dual species community with *F. nucleatum* or *S. gordonii*. The increased ZEB1 expression was associated with enhanced ZEB1 promoter activity. The levels of ZEB1 correlated with increased migration of mesenchymal markers including MMP-9 and vimentin, and with enhanced migration of epithelial cells into matrigel. Knockdown of ZEB1 with siRNA inhibited the *P. gingivalis*-induced enhancement of mesenchymal markers such as vimentin and MMP-9, and migration of epithelial cells into matrigel. In addition, knockdown of ZEB1 with siRNA inhibited the *P. gingivalis*-induced increase in mesenchymal markers and epithelial cell migration. Interestingly, in mice, oral infection with *P. gingivalis* enhanced ZEB1 levels in gingival tissues [54]. *Porphyromonas gingivalis* was also detected intracellularly by antibody staining in biopsies from OSCC [54]. Taken together, FimA-driven ZEB1 expression may, in part, explain a contribution of *P. gingivalis* to OSCC. The *in vitro* and *in vivo* studies listed above showed that *P. gingivalis* may contribute to a mesenchymal phenotype driving the progression of cancer in co-operation with other oral bacteria.

### Porphyromonas gingivalis activates metalloproteinase-9 and interleukin-8 in cultures of carcinoma cells

While proMMP-9 was secreted continuously from carcinoma SAS cells, *P. gingivalis* infection enhanced expression of the proenzyme and induced the proenzyme to activate MMP-9 in a culture supernatant that promoted invasion of the cells [55]. *Porphyromonas gingivalis* activated the signaling cascade extracellular signal-regulated kinase 1/2 (ERK1/2)-Ets1, p38/Heath-shock-protein 27 (HSP27) and Protease activated receptor 2 (PAR2)/nuclear factor kappa-light-chain-enhancer of activated B cells (NF-ĸB) pathways to produce proMMP-9 expression. The gingipain proteases (Arg- and Lys-gingipains) of *P. gingivalis* subsequently activated the proenzyme, which promoted cellular invasion and metastatic abilities of cell lines derived from OSCC. ProMMP-9 was produced by highly invasive/metastatic cells but not by cells with low invasiveness. In contrast, *F. nucleatum* did not perform such activities. Inhibitors of SAS cells reduced proMMP-9 over-expression and cellular tumorigenic invasion. In addition, *P. gingivalis* mutants that lacked gingipain proteases, failed to activate MMP-9. Accordingly, these findings, based on disruption of normal cell signaling, indicated perhaps a new mechanism involved in the progression and metastasis of OSCC associated with periodontitis. Further, this investigation highlighted the increase of HSP27 in oral cancer cells infected with *P. gingivalis* and HSP27’s role in the activation of MMP9 in infected cells [55]. HSP27 is over-expressed in many cancer cell types leading to poor prognosis [56,57]. Hence, HSP27 was actively investigated as a viable therapeutic target in cancer therapies using anti-sense oligonucleotides and/or pharmacological inhibitors [56,58,59]. A recent study showed for the first time phosphorylation of HSP27 by a bacterial effector – *P. gingivalis*-NDK – which confers an anti-apoptotic phenotype to human primary oral epithelial cells [60]. HSP27 may be an essential target to promote clearance of *P. gingivalis* in the oral mucosa. Further, NDK-HSP27 interaction could point to targeted therapeutic strategies against cancers recently associated with *P. gingivalis*.

Exposure to *P. gingivalis* increased the invasive ability of cells derived from OSCC such as OSC-20 and SAS by upregulating interleukin 8 (IL-8) and matrix metalloproteinases, particularly MMP-1 and MMP-2 [61]. This was in contrast to SCC-25 cells that did not exhibit changes in their cellular invasive properties or low expression levels of MMPs. The finding corresponded with the IL-8 secretion, which was substantially enhanced in the OSC-20 and SAS cells but not in SCC-25 cells after infection with *P. gingivalis*. However, when IL-8 was directly applied upon SCC-25 cells, these cells’ invasive ability and MMP level increased significantly. In contrast, downregulation of IL-8 in *P. gingivalis*-infected OSC-20 and SAS cells reduced their invasive potentials and levels of MMP. Thus, *P. gingivalis* increased the invasiveness of OSCC cells by IL-8-dependent upregulation of matrix metalloproteinases. Interestingly, MMP-9 activation and spreading of OSCC could be prevented by inhibitors such as apple polyphenol (AP), hop bracht polyphenol (HBP) and high-molecular fractions of HBP (HMW-HBP). These inhibitors could be candidate cytostatic agents to prevent such cancerous invasion [62].

### Porphyromonas gingivalis accelerates cell cycling and suppresses apoptosis in cultures of gingival epithelial cells

Bacteria regulate the cell cycle of their host cells to be able to survive and express their virulence factors within the host. *Porphyromonas gingivalis* induced changes in the level and phosphorylation status of proteins that display multilevel control on the eukaryotic cell cycle involving cyclins, p53 and Phosphatidylinositol-4,5-bisphosphate 3-kinase (P13K) [63]. The increased proliferation rate of infected human primary gingival epithelial cells was followed by accelerated progression of the S-phase. Also Pan et al. [64] demonstrated that *P. gingivalis* affects the cell cycle of human gingival epithelial cells by promoting the G1/S transition, which implies upregulation of cyclin D and cyclin E. Accordingly, contribution from cell proliferation-enhancing bacteria to carcinogenesis should not be overlooked, although the latter is a protracted multistage and multifactorial process.

In primary cultures of short-term gingival epithelial cells, *P. gingivalis* inhibited apoptosis by upregulating the anti-apoptotic molecule Bcl-2 and downregulating the pro-apoptotic Bad protein [65]. Yilmaz et al. [66] reported that *P. gingivalis* blocks apoptosis and increases survival of primary gingival epithelial cells through activation of the pPI3K/Akt survival pathway. Later, Mao et al. [67] suggested that one mechanism by which *P. gingivalis* can block apoptosis in gingival epithelial cells is through manipulation of the P13K/Akt and JAK/Stat pathway that controls intrinsic mitochondrial cell death pathways. In addition, sequestration of pro-apoptotic Bad, a member of the Bcl-2 family, through Akt, has been demonstrated [68]. Akt is a major component of anti-apoptotic pathways that are stimulated by *P. gingivalis*. The latter can also inhibit apoptosis in primary gingival epithelial cells induced by a danger molecule, i.e. ATP by ligation of P2X_7_ receptors [69]. Agents that can impede apoptosis may promote the build-up of cancerous cells [26]. In addition, miRNA-203 from *P. gingivalis* suppresses SOC6 that has an important role in modulating mitochondrial dynamics and subsequent apoptotic events [70,71]. Regulation of the cell cycle by miR-203 may affect the pathological expression in some carcinomas [72]. Accordingly, suppression of mitochondrial-dependent apoptosis may be an important stratagem for the survival of *P. gingivalis* in periodontal tissues and a key feature of its pathogenicity. In addition, spread of this bacterium to other cells could be achieved by protection from the immune system through reduced inflammation in epithelial tissues [51,73].

### Porphyromonas gingivalis induced cell cycle arrest and increased autophagy, but had no effect on apoptosis in oral cancer cells

Although several published studies have dealt with the effect of *P. gingivalis* on oral epithelial cells, little is known about its effect on cancer cells. When oral cancer cells were invaded by *P. gingivalis* FDC 381, proliferation of the cells were inhibited through arrest of the G1 cell cycle. There was no effect on apoptosis, but macro autophagy was increased [74]. It was suggested that oral cancer cells promote macro autophagy as an adaptive mechanism to invasion of *P. gingivalis* within the tumor, serving as a survival mechanism that limits bacterial toxicity. The expression of cyclin D1 and cyclin-dependent kinase 4 (cdk4) was reduced in the oral cancer cells, while the Cdk inhibitor – p21- was upregulated, compared to non-infected controls. The increased autophagy response probably contributed to the suppressed proliferation. This response was activated by formation of reactive oxygen species (ROS). Researchers have also found that *P. gingivalis* increases cell proliferation by accelerating cell cycling or by pro-survival signaling via modulation of apoptosis [74]. The reported discrepancies in these molecular events are likely results of studies using distinct host model systems (i.e. infection of normal epithelial cells versus cancer cells) and could reveal key information as to the tumorigenic conversion. Consistent with this view, *P. gingivalis* infection of human primary oral epithelial cells has been shown to modulate and inhibit both mitochondrion- and membrane (NADPH-oxidase)-derived ROS to secure successful intracellular growth and survival [75,76]. Moreover, *P. gingivalis* employs a selective form of autophagy (pro-bacterial autophagy) that is rich in ER-networks in order to replicate and persist in the human primary epithelial cells [77].

### Porphyromonas gingivalis promotes distant metastasis and chemoresistance to anti-cancer agents

Sustained infection with *P. gingivalis* promoted distant metastasis of oral cancer to the lungs together with development of resistance to anti-cancer agents [78]. This was because tumor xenografts of *P. gingivalis*-infected cells from OSCC exhibited a higher resistance to the chemotherapeutic agent Taxol through Notch intracellular domain (NICD) 1 activation. It suggested that *P. gingivalis* might have a role in the development of chemoresistance towards OSCC. Similarly, when OSCC cells were exposed repeatedly with *P. gingivalis* for 5 weeks, resistance to the chemotherapeutic agent Taxol was developed [48]. Interestingly, targeting Notch signaling pathways may be used to overcome drug resistance to cancer therapy, preferably in combination with conventional cytotoxic chemotherapy [78,79].

### Porphyromonas gingivalis accelerates proliferation of oral tumor cells by affecting gene expression of defensins and noncanonical activation of β-catenin

Hoppe et al. [80] showed that oral pathogens such as *P. gingivalis* and *Aggregatibacter actinomycetemcomitans* increased proliferation properties of oral tumor cells by affecting gene expression of human defensins. While incubation of oral tumor cells with *P. gingivalis* and human α-defensins led to increased cell proliferation, *A. actinomycetemcomitans* caused increased cell death. Accordingly, these periodontal pathogens had opposite primary effects on the proliferation of oral tumor cells, but both caused similar secondary effects on the proliferation rate by modifying expression levels of oncogenic relevant α-defensin genes. It was suggested that anti-microbial peptides could function as a molecular link between tumorigenesis and infection since human defensins affect cell proliferation via Epidermal Growth Factor Receptor (EGFR)-dependent signaling.

Zhou et al. [81] reported that noncanonical activation of β-catenin signaling by gingipain-dependent proteolytic processing might be a potential mechanism for *P. gingivalis* to contribute to tumorigenesis. It could be so, because noncanonical activation of β-catenin and disassociation of the β-catenin destruction complex by gingipain might induce a proliferative phenotype. This could be a novel mechanism for *P. gingivalis* to contribute to disruption of oral tissue homeostasis by proteolytic processing.

### Porphyromonas gingivalis converts ethanol to acetaldehyde

Acetaldehyde (ACH), which is the first metabolite of alcohol, is a group 1 carcinogen to humans when associated with intake of alcoholic beverages [82]. Subjects with poor oral hygiene had a 2-fold higher *in vitro* salivary ACH production from ethanol compared to individuals with good oral hygiene [83,84]. *Porphyromonas gingivalis* converts ethanol to acetaldehyde at levels capable of inducing DNA damage, mutagenesis, and hyperproliferation of the epithelium [50]. This may partially help explain why heavy drinking and some cancers are related [23].

### Possible role of Porphyromonas gingivalis epigenetic modifications for inflammation

Chronic periodontitis is one of the most common inflammatory diseases in humans. Stimulation and permanency of inflammation are regulated by complex mechanisms among which epigenetic pathways have received special attention. Epigenetic modification includes chemical changes in DNA and associated proteins. This can lead to remodeling of the chromatin and activation/inactivation of gene transcription [85]. Such changes can contribute to cancer and autoimmune and inflammatory diseases including periodontitis. Both DNA and histone modifications, two major epigenetic regulations, have been detected in periodontitis, and gene expression here can be affected by DNA methylation [86]. Indeed, aberrant DNA methylation has been found in gingival tissue [87,88]. Recently, it was shown that *P. gingivalis* LPS markedly regulates genes involved in epigenetic mechanisms [85].

### Potential role of peptidyl arginine deiminases and protein citrullination in cancer pathogenesis

*Porphyromonas gingivalis* is currently known as the only pathogen that produces peptidyl-arginine deiminase (PAD) [89]. This enzyme modifies both bacterial and host proteins by deiminating arginine residues in proteins and peptides, converting them into citrulline [18,90–92]. Protein citrullination deregulates the host’s inflammatory signaling network by changing the spatial arrangement of the original 3D-structure and function of the protein [89,93]. The host has also intrinsic sources of citrullination through genes coding for a family of five calcium-dependent enzymes named peptidyl-arginine deiminases (PAD 1, 2, 3, 4/5 and 6), which are quite similar but not identical to PPAD. The host PADs have been linked to various human and animal cancers [94–97]. Thus, Kholia et al. [96] reported that the expression level of PAD2 and PAD4 and the deimination of cytoskeletal actin were increased during stimulation of prostate cancer cells (PC3) with microvesiculate, which is involved in the progression of cancer. Pharmacological inhibition of the PAD enzyme activity using the pan-PAD-inhibitor chloramidine (Cl-am) significantly reduced the release of microvesicles and abrogated the deimination of cytoskeletal actin. PAD4, which is overexpressed in several types of invasive carcinomas [94,98,99], and possibly PAD2, seem to have important roles in the progression of tumors. This may also relate to PAD. Inhibitors of PAD can suppress both tumor progression and inflammatory symptoms [97].

## *Porphyromonas gingivalis* and esophageal squamous carcinoma

Esophageal cancer is the eighth most frequent cancer and the sixth leading cause of cancer death worldwide [50]. There had not been convincing evidence of specific microbiological agents in esophageal squamous carcinoma (ESCC) until Gao et al. [50] detected with immune histochemistry a high frequency of *P. gingivalis* in ESCC, i.e. 61% in ESCC and 12% in adjacent tissues, while it was undetected in the normal esophageal mucosa. A similar distribution of lysine-specific gingipain and 16S rDNA of *P. gingivalis* was detected. Median serum levels of IgA and IgG against *P. gingivalis* were significantly higher in ESCC than in esophagitis and healthy controls [39]. Conventional serum markers for ESCC such as squamous cell carcinoma antigen (SCCA), cardioembryonic antigen (CEA), CYFRA21-1 and carbohydrate antigen (CA) 19–9 did not exhibit adequate sensitivity and specificity for early detection and progression of ESCC [39]. IgA for diagnosing early ESCC proved to be much better than IgG (54.54% versus 20.45%) [39]. Furthermore, high serum levels of IgA or IgG against *P. gingivalis* were correlated with a worse prognosis in ESCC patients, particularly in those with stage 0–II or negative lymph node metastasis. Patients with both high IgA and IgG had the worst prognosis. Therefore, combining IgA and IgG may improve diagnosis and lead to better prognosis. The findings of this study implied that *P. gingivalis* might be involved in the pathogenesis of ESCC and that combination of several biomarkers is better than any single biomarker for its diagnosis. Therefore, *P. gingivalis* serum biomarkers could be important for detection of ESCC at an early stage and may improve diagnostic and prognostic performance.

Peters et al. [100] found in a prospective study nested in two cohorts that the oral microbiome, recorded by 16S rRNA gene sequencing of mouthwash samples, may reflect a prospective risk for esophageal cancers. While *P. gingivalis* abundance tended towards a higher risk for ESCC, *Tannerella forsythia* was associated with a higher risk of esophageal adenocarcinoma (EAC). Other bacteria such as *Neisseria* and *S*. *pneumoniae* were associated with lower EAC risk. *Porphyromonas gingivalis* was also associated with ESCC lymph node metastasis and reduced survival time [50].

In a study by Yuan et al. [101], *P. gingivalis* was detected preferentially and frequently in esophageal cancer and dysplasia of the esophagus, but rarely in matching non-cancerous portions. This bacterium was quite low or absent in cancers from the cardia or remaining stomach. The variance was ascribed to less tolerance of *P. gingivalis* to acidic pH values.

## *Porphyromonas gingivalis* in head and neck (aerodigestive tract) squamous cell carcinoma

Head and neck squamous cell carcinoma (HNSCC) is the sixth most common cancer world-wide with a 50–60% survival rate [102,103]. Using samples from HNSCC, Utispan et al. [104] found that *P. gingivalis* LPS-activated macrophage-conditioned medium promoted invasion of primary and metastatic cell lines that had been derived from stage II, III and IV HNSCO. Proliferation of HN4 cells (cell line from HNSCC) was inhibited. The tumorigenic invasion may have been mediated by induced macrophage Nitric oxide (NO) generation through the EGFR signaling pathway, but needs further study.

## *Porphyromonas gingivalis* and precancerous gastric and colon lesions

Among patients who undergo gastroscopy with biopsy for clinical indications, approximately one in 19 with dysplasia will develop gastric cancer within 20 years [105]. In individuals with high levels of periodontal disease, periodontal pathogens detected in plaque were related to an increased risk for precancerous gastric lesions independent of confounding factors, although no consistent association between DNA levels of oral pathogens and presence of gastric precancerous lesions could be established [106]. Sun et al. [107] found that the burden of periodontal pathogens and bacterial diversity in the oral cavity were important factors contributing to a potentially increased development of precancerous gastric cancer. However, gastric samples for direct microbial analysis were not taken.

## *Porphyromonas gingivalis* and pancreatic cancer

Globally, about 338,000 people had pancreatic cancer in 2012, which made it the 11th most common cancer with the highest incidence and mortality rates found in developed countries [108]. Positive associations between the salivary microbiota of patients with pancreatic cancer and chronic pancreatitis have been reported [109]. Periodontal disease and *P. gingivalis* may be important to the development of pancreatic cancer [110,111]. Periodontopathogens may contribute by acting alone or together with other pancreatic cancer risk factors such as smoking, obesity and the *ABO* genetic variant [110]. A prospective nested case-control study recently demonstrated that carriage of *P. gingivalis* and *A. actinomycetemcomitans* together with decreased relative abundance of the phylum Fusobacteria and its genus *Leptotrichia* were related to an increased risk of pancreatic cancer [38]. The study was based on oral mouthwash samples from 361 men and women with incident adenocarcinoma of pancreas and 371 matched controls. The participants were monitored for almost a decade to assess those who developed pancreatic cancer. Evidence was provided that the oral microbiota may play a role in the etiology of pancreatic cancer irrespective of potential confounders. Thus, participants with *P. gingivalis* had a 59% higher risk of developing cancer than those without, and patients with *A. actinomycetemcomitans* had a 50% risk. Noticeable was that the oral microbial dysbiosis (*P. gingivalis* and *A. actinomycetemcomitans*) preceded cancer development [38]. In a European nested case-control study involving 404 pancreatic cancer cases and 410 controls, high IgG antibody levels (>200 ng/mL) to *P. gingivalis* ATCC 53978, which is strongly associated with destruction of the periodontium, were associated with a > 2-fold increased risk to pancreatic cancer after adjusting for known risk factors, compared to those with lower antibody levels (≤200 ng/mL) [111]. In addition, subjects with high levels of antibodies to common oral bacteria had a 45% lower risk of pancreatic cancer compared to those with lower antibody levels. It is noteworthy that antibodies to *P. gingivalis* ATCC 53978 were the best antibody marker for a high bacterial load and aggressive periodontitis. In addition, Ahn et al. [24] found in the NHANES III study that clinically defined periodontitis was associated with a 3-fold increased risk of orodigestive cancer mortality. It seems plausible that *P. gingivalis* can reach the pancreas and contribute in pancreatic carcinogenesis.

## Concluding remarks

There has been an increasing appreciation of a direct relationship between oral infection/inflammation and cancer. The most convincing evidence for such a relationship applies to periodontitis and orodigestive cancers. This relationship exists even after confounding factors, e.g. smoking, body mass index, and socioeconomic status have been accounted for. The underlying molecular mechanisms have not yet been fully clarified, but murine models of periodontitis-associated carcinogenesis have recently shown that long-term chronic bacterial infection promotes OSCC through direct interaction with cancerous and pre-cancerous oral epithelial cells via toll-like receptors. Members of the oral microbiota may directly stimulate OSCC proliferation and induce expression of key molecules (e.g. NF-kB, IL-6-STAT3, cyclin D1, MMP-9, and the bacterial gingipains) that are implicated in tumorigenesis. It seems very possible that *P. gingivalis* in conjunction with established risk factors e.g. alcohol consumption, may play a role in increased cancer development. It has been suggested that in the presence of risk factors such as alcohol and tobacco abuse, the commensal oral microbiota may act synergistically in the oral cancer pathogenesis [112]. Although these well recognized carcinogenic factors certainly can contribute, they cannot alone explain the large amount of cancer cases developing each year. Several other possible mechanisms exerted by *P. gingivalis*, a keystone pathogen in periodontitis, are discussed in the current review. *Porphyromonas gingivalis* is probably not the only carcinogenic agent in the oral microbiota where coinfection with other bacteria, e.g. *F. nucleatum* and *Prevotella intermedia* occurs. Not every person either carries *P. gingivalis* that may induce cancer, irrespective of periodontitis and may be associated with worse prognosis. Indeed, serum antibodies to *P. gingivalis* may be used to improve cancer diagnosis and prognosis. The fact that pancreatic and gastric cancers can be associated with *P. gingivalis* suggests that oral microorganisms, bacterially-secreted effectors, inflammatory cells and mediators travel with saliva and blood to distant sites and induce systemic carcinogenic effects. This is also supported by the finding that a greater serum level of *P. gingivalis* IgG can be associated with orodigestive cancer. It is compelling to propose that there may be a direct relationship between *P. gingivalis* and orodigestive cancers, where the contribution to carcinogenesis may be due to secondary intrusion of the oral microorganism outside of its primary location (oral cavity), yet still within anatomically continuous regions. More molecular epidemiological and mechanistic studies with larger cohorts and better controls will be important to answer these emerging questions.
